# CiteSpace-based visual analysis on transcutaneous electrical acupoint stimulation of clinical randomized controlled trial studies and its mechanism on perioperative disorders

**DOI:** 10.1097/MD.0000000000039893

**Published:** 2024-10-11

**Authors:** Mengqi Li, Xiaobo Jiang, Xiangmu Gai, Mengyao Dai, Mengyuan Li, Yanxin Wang, Hongfeng Wang

**Affiliations:** a College of Acupuncture and Tuina, Changchun University of Chinese Medicine, Changchun, Jilin, China; b Northeast Asia Institute of Traditional Chinese Medicine, Changchun University of Chinese Medicine, Changchun, Jilin, China; c Department of Cardiovascular Rehabilitation, The Third Clinical Affiliated Hospital of Changchun University of Chinese Medicine, Changchun, Jilin, China.

**Keywords:** CiteSpace, mechanism, randomized controlled trial, TEAS, visual analysis

## Abstract

To systematically present an overview of randomized controlled trials on transcutaneous electrical acupoint stimulation (TEAS) using bibliometric methods, and describe the role and mechanisms of TEAS in most prevalent diseases. Relevant literature was searched in China National Knowledge Infrastructure, Wanfang Data, VIP, SinoMed, PubMed, and Web of Science. The literature was imported and screened into NoteExpress, screened according to inclusion and exclusion criteria, and analyzed using Excel and CiteSpace 6.3R1 software. A total of 1296 documents were included. The number of publications increased annually after 2012. Junlu Wang was the most prolific author. The main research institutions were Peking University, The Third Affiliated Hospital of Zhejiang Chinese Medical University, Shuguang Hospital, and Tongde Hospital of Zhejiang Province. The research hotspots in this field include perioperative care, cancer, pain management, and stroke, primarily focusing on analgesia, immune enhancement, antihypertension, and reduction of gastrointestinal disorders. The main regulatory mechanisms of TEAS include the control of inflammation, oxidative stress, and regulation of the autonomic nervous system. TEAS is most widely used in the elderly, with PC6, ST36, and LI4 being the most frequently studied acupoints in clinical randomized controlled trials. The concept of accelerated rehabilitation is gradually being applied to TEAS, representing an emerging trend for future development. Clinical research on TEAS is rapidly developing, with a focus on applications in cancer and perioperative care. Future research should expand collaboration and conduct high-level clinical and mechanistic studies, which will contribute to the development of standardized protocols and clinical practice.

## 1. Introduction

The continuous development of clinical interventions has witnessed the fusion of traditional therapeutic approaches with advanced technologies. Transcutaneous electrical acupoint stimulation (TEAS), an innovative modality combining China’s traditional meridian and acupoint theories with transcutaneous electrical nerve stimulation technology, epitomizes this fusion. TEAS has been applied to a range of diseases, from chronic pain syndromes to postoperative recovery and even as an adjunct to psychiatric treatment. Its advantages include painless treatment, reduced labor costs, and minimized risk of needle breakage, making it an important complementary and alternative medicine method.

The growing literature on randomized controlled trials (RCTs) of TEAS provides a powerful source of evidence for meta-analyses and systematic reviews, essential for guiding clinical practice and patient care protocols. Systematic reviews of RCTs are crucial to validate and consolidate findings on the effectiveness of this treatment modality and to establish standardized protocols. Most current research reviews build on traditional reviews, meta-analyses, and systematic evaluations of the efficacy and safety of TEAS for clinical disease treatment.^[1,2]^ However, in addition to outcome data from clinical studies, patterns of research collaborations, thematic focus, and temporal development offer valuable insights that can shape the trajectory of future research and implementation.^[3,4]^

The purpose of this paper is to leverage the bibliometric capabilities of CiteSpace to visualize and analyze RCTs investigating TEAS. Through CiteSpace, this study will synthesize data related to publication trends, coauthor networks, institutional collaborations, and keyword research and examine the evolution of the field over time. The resulting visualization network will illustrate the depth and breadth of TEAS research, highlighting research hotspots, influential work, and emerging trends while providing a comprehensive overview of the role and mechanisms of TEAS in perioperative diseases.

## 2. Information and methodology

### 2.1. Retrieval strategy

Using search terms such as “transcutaneous acupoint electrical stimulation,” “transcutaneous electrical stimulation,” and “transcutaneous nerve electrical stimulation,” we searched the China Knowledge, Wanfang, Wipro, SinoMed, PubMed, and Web of Science databases to retrieve all Chinese and English literature up to March 21, 2024, on TEAS. The English search formula was based on Web of Science, for example: transcutaneous acupoint electrical stimulation (topic) or TEAS (topic) or transcutaneous electrical stimulation of acupoints (topic) or transcutaneous electrical acupuncture point stimulation (topic) or transcutaneous electrical nerve stimulation on acupoints (topic) or transcutaneous electric nerve stimulation (topic).

### 2.2. Inclusion criteria

(I) Study type: RCT; (II) study population: unlimited; (III) interventions and controls: interventions based on TEAS, including literature on TEAS in combination with other modalities of treatment, such as traditional Chinese medicine, Western medicine, or other acupuncture modalities; and (IV) completeness of outcome indicators.

### 2.3. Literature exclusion criteria

(I) Duplicate publications (only the latest published literature is retained); (II) full text not available; (III) protocols, systematic evaluations, reviews, conference papers, patents, scientific and technological achievements, scientific and technological reports, and guidelines; (IV) animal experiments; (V) literature with incomplete information on the year, authors, institutions, keywords, etc; and (VI) other obviously unrelated studies.

### 2.4. Screening and extraction of literature

Two researchers (Mengqi Li and Xiangmu Gai) independently conducted database screening and literature data extraction. They imported the literature obtained from each database into NoteExpress (V3.5), eliminated duplicates, read the titles and abstracts to eliminate literature that did not meet the inclusion and exclusion criteria for initial screening, and read the full text for further screening to confirm the final inclusion of the literature. In case of disagreement between the 2 researchers during the screening process and data extraction, a third party (Xiaobo Jiang) was assigned to make a judgment. The literature data were then exported from NoteExpress 3.5 in Refworks format and saved in “.txt” format. The titles, authors, institutions, and keywords of the English literature were translated manually and then exported in Refworks format, ensuring accuracy in authors’ names and institutions during the translation process. After confirming the completeness of the key information, such as titles, authors, institutions, and keywords, we cleaned the data according to the following method.

#### 2.4.1. Regulating the names of institutions

For institutions with different subordinate departments, the names of subordinate departments were uniformly deleted to retain only the names of the first-level institutions. For example, “China Academy of Chinese Medical Sciences Acupuncture and Moxibustion Research Institute” and “China Academy of Chinese Medical Sciences Basic Theory of Traditional Chinese Medicine Research” were unified and revised to “China Academy of Chinese Medical Sciences.” The former names of institutions were updated to their current standardized names, such as “Zhejiang College of Traditional Chinese Medicine,” which was unified as “Zhejiang Chinese Medical University.” Institutions with multiple names were standardized to 1 name, such as “University of Science and Technology of China affiliated hospitals” and “Anhui Provincial Hospital,” which were unified as “Anhui Provincial Hospital.”

#### 2.4.2. Standardized terminology translation

To ensure the accuracy and correctness of English translations of terminology, researchers first conducted frequency statistics on key information such as authors, institutions, and keywords in NoteExpress 3.5. Using a combination of DeepL software and manual translation, they translated the key information and replaced all key terms with English using the NoteExpress 3.5 replacement function.

#### 2.4.3. Merge keywords

Keywords that are significant for graph interpretation, as well as keywords with the same meaning but different expressions, should be unified into standardized terms or common expressions. For example, “transcutaneous electrical acupoint stimulation” and “TEAS” should be uniformly changed to “transcutaneous electrical acupoint stimulation.”

### 2.5. Software setup

Input the final data into CiteSpace 6.3R1, convert the input data into a readable form, and store it in the project file for data analysis. The Time Slicing in the software is set to “January 1988–December 2024,” and all other settings are set as default, except for Pruning—Pathfinder and Pruning the merged network algorithms for data cropping when analyzing keywords.

## 3. Results

### 3.1. Analysis of publications

**Figure 1.**
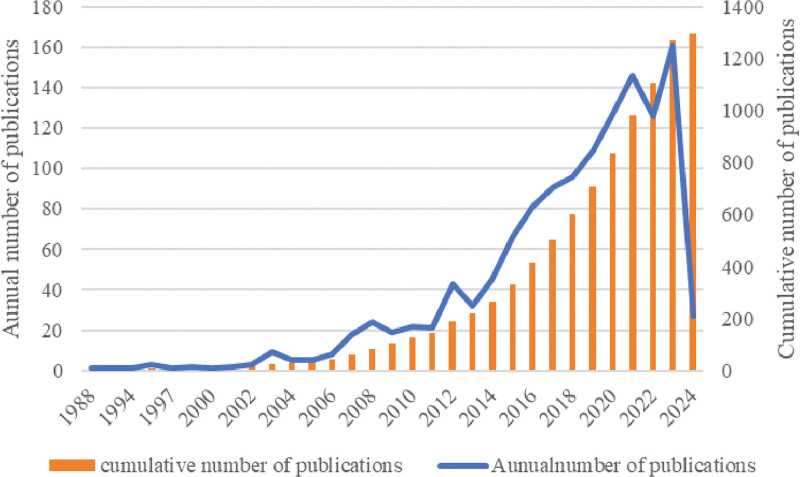
The states of annual publications.

A total of 7084 articles were retrieved, including 5610 articles in Chinese and 1474 articles in English. According to the inclusion and exclusion criteria, 1296 articles were included, of which 134 were in English. The number of articles published in RCTs of TEAS showed a slow increasing trend from 1988 to 2010, with a more pronounced increase after 2012. The number of articles published in TEAS RCTs reached a peak in 2023, with 161 articles, but has decreased since 2022. Overall, the trend of articles published has remained stable. See Figure 1.

The included literature was published in 492 journals, including 395 Chinese journals and 97 English journals. Based on *the 2023 edition of the Chinese Core Periodicals Abstract* published by Peking University and *the 2023 edition of the Chinese Science and Technology Journal Citation Report (Core Edition*) published by the China Institute of Scientific and Technological Information, 174 of the 395 Chinese publications belonged to the core of Chinese/Science and Technology journals, accounting for 35.37%. Table 1 shows the top 10 journals with the number of publications. The total impact factor of English journals was 298.11, with an average impact factor of 3.07 and the highest impact factor of 11.9 (*Psychiatry and Clinical Neurosciences*). The journal *Medicin*e had the highest output of clinical RCTs in TEAS among English journals, accounting for 5.15% of the total English publications.

**Table 1:** Top 10 journals with the number of publications.

Rank	Journal	Number of publications	Chinese core journals
1	Chinese Acupuncture and Moxibustion	54	Yes
2	Shanghai Journal of Acupuncture and Moxibustion	39	No
3	Chinese Journal of Anesthesiology	29	Yes
4	Acupuncture research	27	Yes
5	Journal of Clinical Anesthesiology	25	Yes
6	Zhejiang Journal of Traditional Chinese Medicine	25	No
7	Chinese Journal of Integrated Traditional and Western Medicine	18	Yes
8	Medical and Health	18	No
9	Journal of Emergency in Traditional Chinese Medicine	17	No
10	Modern Journal of Integrated Traditional Chinese and Western Medicine	16	No

### 3.2. Author analysis

The author collaboration co-occurrence network is used to identify researchers in the field of study and the collaboration between individual researchers. This study involves a total of 4024 authors, with Junlu Wang having the highest number of publications at 27. According to Price law,^[5]^ if the total number of publications by core authors is >50%, a core author group within the field has been formed. In this study, M=0.749×√27≈4 articles. Authors with more than 4 publications were considered core authors, totaling 162, with a combined total of 961 publications, accounting for 74.15% of the total included literature, indicating the formation of a core group of authors in the field of clinical RCTs in TEAS.

**Figure 2.**
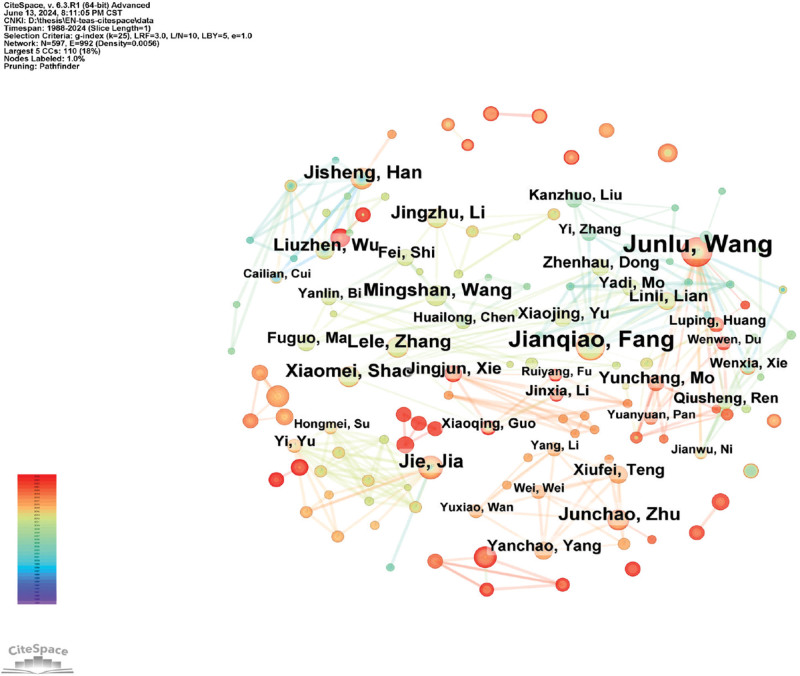
Collaborative co-occurrence network of core authors. The size of the nodes indicates the number of articles published by authors, and the connecting lines between nodes indicate the cooperation between authors.

The network co-occurrence of the core authors, visualized using CiteSpace, shown in Figure 2, included 597 nodes and 992 connections, with a network density of 0.0056. A collaborative network centered around Junlu Wang and Jianqiao Fang was identified for clinical RCTs on TEAS. However, the network density was sparse, and the connections between nodes were thin, indicating that while there was some collaboration among authors, the overall cooperation between authors needs to be further strengthened.

### 3.3. Institutional analysis

**Figure 3.**
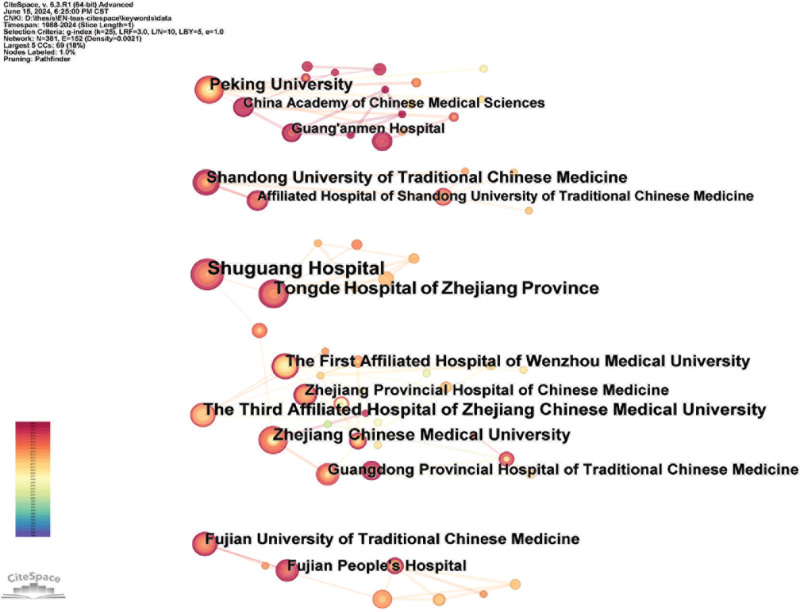
Collaborative co-occurrence network of institutions.

A total of 1051 research institutions participated in the clinical randomized controlled study of TEAS. CiteSpace was used to form an institutional cooperative network relationship map (shown in Fig. 3). Peking University, The Third Affiliated Hospital of Zhejiang Chinese Medical University, Shuguang Hospital, and Tongde Hospital of Zhejiang Province were the main institutions involved in the study. The research institutions are primarily concentrated in 5 regions: Beijing, Zhejiang, Fujian, Shandong, and Shanghai. The institutions are relatively decentralized, mainly manifested in the cooperation between universities and their affiliated hospitals, such as Zhejiang Chinese Medical University and The Third Affiliated Hospital of Zhejiang Chinese Medical University. There is less cooperation among cross-regional institutions.

### 3.4. Keyword analysis

#### 3.4.1. Keyword centrality analysis

Keywords briefly summarize the content of a paper and its core areas of focus, contributing to a broader understanding of the subject area and highlighting trends, gaps, and connections in the research. In keyword analysis, the frequency and centrality of keyword occurrences reflect the prominence and trend of research. Generally, high frequency of keywords and large centrality of nodes represent the heat of the research.^[6]^

Table 2 shows the centrality of keywords with a frequency >30 occurrences. The results indicate that keywords with high frequency and centrality >0.1 include *transcutaneous electrical acupoint stimulation*, *transcutaneous electrical nerve stimulation*, *acupoint*, *cancer*, *acupuncture and moxibustion*, *the elderly*, *cerebral apoplexy*, *transcutaneous electrical stimulation*, *stress response*, *pain*, and *ST36*. Keywords such as *transcutaneous electrical acupoint stimulation*, *transcutaneous electrical nerve stimulation*, *acupuncture and moxibustion*, and *transcutaneous electrical stimulation* belong to the same therapeutic modality. The diseases related to the research hotspots of TEAS are mainly focused on cancer, cerebral apoplexy, and pain. The elderly are the most widely studied group for TEAS, and PC6 and ST36 are the hotspots of research in clinical randomized controlled studies of TEAS.

**Table 2:** Keyword centrality (keyword frequency > 30 times).

Arrange in order	Frequency	Centrality	Year of first occurrence	Keyword
1	897	0.61	1996	Transcutaneous electrical acupoint stimulation
2	137	0.16	1988	Transcutaneous electrical nerve stimulation
3	110	0.17	1996	Acupoint
4	71	0.13	2009	Cancer
5	68	1.08	1988	Acupuncture and moxibustion
6	65	0.1	2007	The elderly
7	61	0.14	2004	Cerebral apoplexy
8	50	0.04	2012	Electric stimulation therapy
9	44	0.02	2010	Postoperative nausea and vomiting
10	44	0.14	1996	Transcutaneous electrical stimulation
11	39	0.06	2005	Analgesia
12	39	0.03	2003	PC6
13	38	0.14	2007	Stress response
14	36	0.32	1988	pain
15	32	0.06	2013	General anesthetic
16	32	0.11	2008	ST36
17	31	0.05	2009	Postoperative analgesia

#### 3.4.2. Keyword co-occurrence analysis

**Figure 4.**
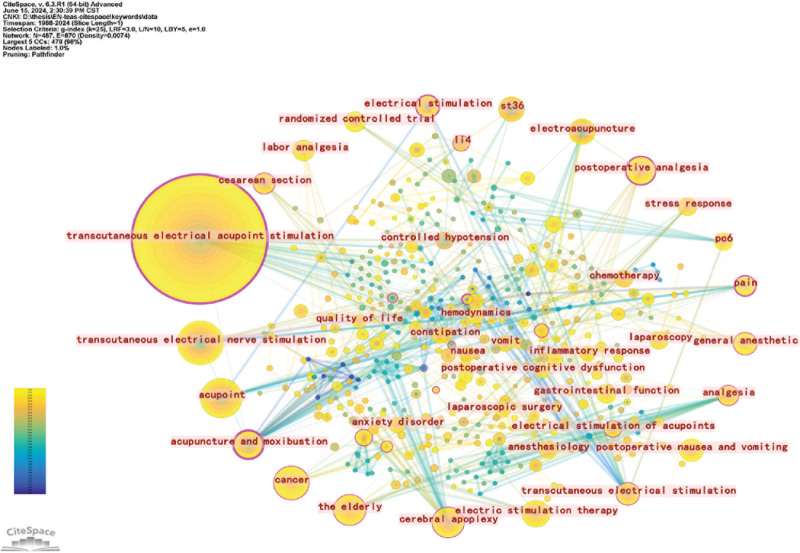
Co-occurrence network of keywords. Node size indicates the frequency of keyword occurrence, and the connecting line between nodes indicates the connection between keywords. Different shades represent different years: cooler shades (blue) represent years closer to 1988, and warmer shades (yellow) represent years closer to 2024.

Figure 4 exhibits the keyword co-occurrence mapping: the figure shows a total of 487 keywords, with 870 node links and a network density of 0.0074. Keywords with larger nodes in the figure mainly include *transcutaneous electrical acupoint stimulation*, represented by *acupuncture therapy*, *cancer*, *pain*, *cerebral apoplexy*, represented by disease types, and acupoint selection, represented by *PC6* and *ST36*. The result is consistent with the results derived from the keyword centrality analysis.

From the nodes and connectivity of the keywords, the main studies of the cancer disease category are closely related to laparoscopic surgery and other procedures. The warmer tones associated with the cancer node, with most node times skewed after 2018, suggest that cancer-related studies are an emerging research hotspot for TEAS clinical trials at this time. Keywords related to pain also include *analgesia*, *postoperative analgesia*, and *labor analgesia*. These words are related to surgery, and similarly, in the keyword co-occurrence mapping, the keywords related to surgery also include *general anesthetic*, *postoperative nausea*, and *vomiting*.

#### 3.4.3. Keyword clustering analysis

**Figure 5.**
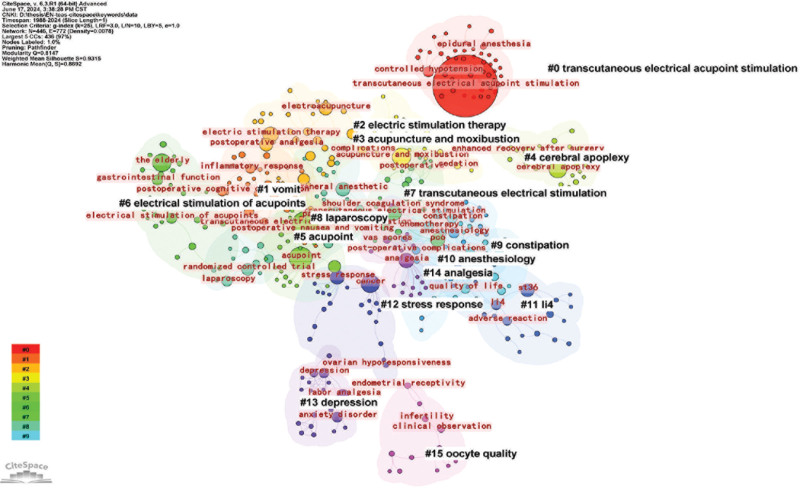
Clustering of keywords.

The keyword clustering analysis using CiteSpace’s Log-Likelihood Ratio algorithm resulted in an *S*-value of 0.9315 and a *Q*-value of 0.8147, indicating the plot’s validity. The results are shown in Figure 5. According to the mapping results, the keywords formed 16 clusters: #0 *transcutaneous electrical acupoint stimulation*, #1 *vomit*, #*2 electric stimulation therapy*, #3 *acupuncture and moxibustion*, #4 *cerebral apoplexy*, #5 *acupoint*, #6 *electrical stimulation of acupoints*, #7 *transcutaneous electrical stimulation*, #8 *laparoscopy*, #9 *constipation*, #10 *anesthesiology*, #11 *LI4*, #12 *stress response*, #13 *depression*, #14 *analgesia*, and #15 *oocyte quality*. The clustering areas were essentially the same for each component.

Clusters #0, #2, #3, #6, #7, #8, and #10 represent treatment measures, while #1, #4, #9, #12, #13, #14, and #15 represent disease types. Clusters #5 and #11 represent acupoints. Keywords appearing more frequently in each cluster are mostly related to surgery and postoperative complications. For example, in the #8 laparoscopy cluster, postsurgical complications such as postoperative pain, constipation, nausea and vomiting, and inflammatory response were frequently observed. Acupuncture points such as LI4 and ST36 are mostly associated with improved quality of life and reduced adverse effects.

#### 3.4.4. Keyword timeline mapping

**Figure 6.**
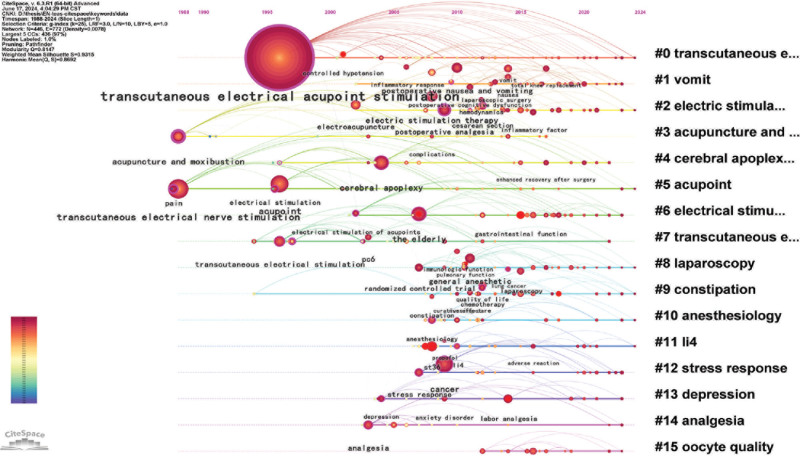
Timeline mapping. Node size indicates the frequency of keyword occurrence, the connecting line between nodes indicates the connection between keywords, and different colors represent different years. The bluer the color, the closer to 1988; the redder the color, the closer to 2024.

CiteSpace was utilized to produce the keyword timeline mapping, and the results are shown in Figure 6. Clusters #0, #2, #3, #6, and #7 are treatment modalities related to TEAS. The names of these modalities mainly existed in the form of a combination of transcutaneous electrical nerve stimulation and #5 acupoint during 1988 to 1995. Since 1996, they have been widely used as stand-alone names in randomized controlled clinical trials.

The timeline for clusters #1 vomit, #9 constipation, #10 anesthesiology, #8 laparoscopy, #12 stress response, and #14 analgesia first appeared between 2005 and 2010. Keywords such as *postoperative nausea and vomiting*, and *postoperative analgesia* appeared at the same time as anesthesiology, suggesting that the rise of TEAS in surgery was centered around 2005 to 2010. Other disorders, such as #4 cerebral apoplexy and #13 depression, appeared between 2000 and 2005 and have continued to be studied to this day.

#### 3.4.5. Keyword with the strongest citation bursts

**Figure 7.**
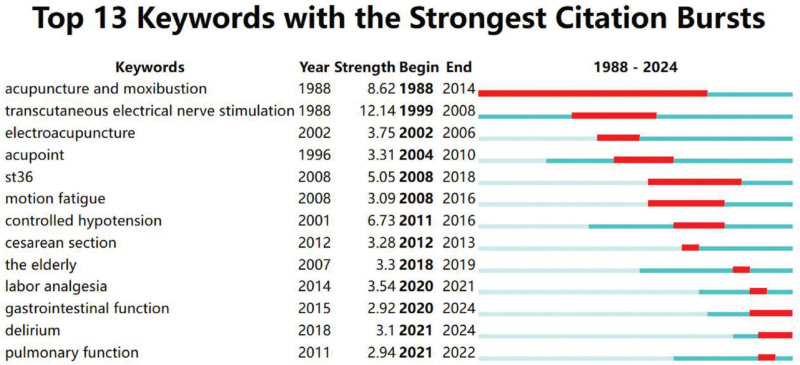
Keywords with the strongest citation bursts mapping.

Figure 7 shows keywords with the strongest citation bursts in RCTs of TEAS during 1988 to 2024. This mapping reflects the periods of different keyword frequency surges and the strength, helping to predict the research hotspots and trends of TEAS in different periods. The results show that the keyword with the strongest citation burst is *transcutaneous electrical nerve stimulation*, with a strength of 12.14, during the time range of 1999 to 2008, which is followed by *acupuncture and moxibustion* and *controlled hypotension*, with a strength of 8.62 and 6.73, and emergence time ranges of 1988 to 2014 and 2011 to 2016, respectively. *Gastrointestinal function*, *delirium*, and *pulmonary function* were the keywords with the highest burst strength in the last 3 years, representing current research hotspots in TEAS RCTs.

## 4. Status of research

Based on the analysis of the annual publication volume, the field was in a state of slow development until 2012, and less attention was paid to the treatment modality of TEAS in clinical studies. Before 1996, TEAS was often used in combination with acupoints and transcutaneous electrical nerve stimulation. However, in the last decade, clinical RCTs of TEAS have rapidly increased, showing a linear upward trend. In recent years, a growing number of studies have emphasized that TEAS can be an alternative to invasive acupuncture therapy, with results comparable to conventional acupuncture therapy.^[7,8]^ Additionally, TEAS has the advantages of being noninvasive, highly maneuverable, and reducing the risk of pain and infection compared to traditional acupuncture, making this new adjunctive treatment modality gradually gain recognition.

Regarding publication venues, Chinese journals remain the primary outlets for TEAS articles. “Chinese Acupuncture & Moxibustion” is the journal that accepts the most articles on TEAS clinical trials and is the most influential in the field of acupuncture. English journals are somewhat less prominent in accepting TEAS trial articles, with only 134 English-language articles published. The highest impact factor among these is 11.9, with “Medicine” being the English-language journal with the highest output. It indicates that the international influence of TEAS needs to be improved, and the relatively low impact factor may be due to the lack of large-scale, double-masked, high-quality, RCTs.

In terms of authorship, Prof Junfeng Wang of The First Affiliated Hospital of Wenzhou Medical University ranks first in the number of publications. A deep dive into Prof Wang literature reveals that his clinical research on TEAS focuses on its perioperative application to explore the improvement of adverse effects through TEAS. Prof Jianqiao Fang of The Third Affiliated Hospital of Zhejiang Chinese Medical University has also published many articles with research directions like those of Prof Wang. Although the number of articles published on TEAS clinical trials is increasing yearly, the number of research institutions conducting TEAS studies is still relatively small, and their cooperation is limited. Research institutions are mainly universities and their affiliated hospitals, with cooperation between university-affiliated hospitals and lower-level hospitals within the same province. However, cooperation among multicenter tertiary hospitals across different provinces is rare. As research on optimizing clinical practice evidence for TEAS continues to deepen, the overall trend of research is positive, and the degree of connection between clinical research institutions is expected to strengthen further.

## 5. Hot diseases and trends in TEAS clinical RCTs

### 5.1. Cancer applications

From the perspectives of keyword frequency, centrality, co-occurrence, and clustering of the included literature, cancer is one of the most common diseases in RCTs of TEAS. Cancer-related keywords have appeared since 2009, and most of the keywords related to cancer in the node timeline of the co-occurrence network are from after 2018. It indicates that cancer and its complications remain one of the hotspots of current TEAS research. According to the *Global Cancer Epidemiology Report 2022*, the top 3 highly prevalent cancers are lung, breast, and colorectal cancers.^[9]^ Meanwhile, lung cancer and lung function are also hot research topics in TEAS trials, which may be significantly associated with the incidence of malignant tumors.

### 5.2. Perioperative applications

Keywords such as *laparoscopic surgery*, *pain*, *hemodynamics*, *propofol* (anesthetic drug), *stress response*, *gastrointestinal function*, and *urinary retention* indicate that TEAS is commonly used to treat various syndromes in the perioperative period, especially with the rise of TEAS in laparoscopic surgery after 2005. The perioperative period refers to the entire process surrounding surgery, including the preoperative, intraoperative, and postoperative phases. Combined with Figure 7, it shows that various syndromes in the perioperative period, such as preoperative anxiety and depression, intraoperative anesthesia status, blood flow, stress reactions, and especially postoperative disorders of gastrointestinal function and elevated blood pressure, are current research hotspots of TEAS.

Clinical research has shown that TEAS has sedative and anxiolytic effects in the preoperative period,^[10]^ provides intraoperative analgesia and organ function protection,^[11,12]^ regulates hemodynamics and stress responses (e.g., blood pressure, tachycardia),^[13,14]^ prevents postoperative nausea, vomiting,^[15]^ and delirium, regulates gastrointestinal function,^[16]^ relieves urinary retention,^[17]^ improves cognitive dysfunction,^[18]^ and offers other benefits.

From the keyword timeline mapping, with the increasing indications of TEAS in the perioperative period, the concept of enhanced recovery after surgery (ERAS) is gradually being applied to TEAS RCTs around 2020. It is an emerging trend in TEAS research. ERAS specifically refers to the clinical practice of integrating evidence-based perioperative measures with optimized clinical pathways to reduce traumatic stress, promote early recovery of organ function, reduce complications, and shorten hospital stays.^[19]^ The ERAS concept is currently being implemented and promoted in various types of surgeries. For example, Jiang et al utilized auricular magnetic bead pressure combined with TEAS to assess the perioperative recovery of patients undergoing radical surgery for thyroid cancer, examining preoperative anxiety, intraoperative medication dosage, and postoperative recovery. They found that acupoint stimulation effectively reduced the dosage of intraoperative analgesic medication, lowered postoperative pain, accelerated postoperative intestinal expulsion, improved the quality of recovery, and sped up patient recovery.^[20]^ It demonstrates that, under the guidance of the ERAS concept, TEAS as an adjunct therapy in the perioperative period can synergistically enhance patient outcomes and optimize clinical pathways.

### 5.3. Stroke applications

From keyword centrality and clustering analysis, it was concluded that stroke is also a significant area for TEAS treatment. Clustering mapping and keyword co-occurrence show that stroke is associated with accelerated surgical rehabilitation and quality of life improvements, as well as addressing emotional dysfunctions such as anxiety and depression. It remains a current direction of research.

## 6. Mechanism of TEAS on perioperative diseases

Among the hot topics studied, TEAS has the greatest focus on perioperative diseases. In most surgeries, especially cancer surgeries, patients suffer from pain, gastrointestinal dysfunction, organ dysfunction, cognitive disorders, decreased immune function, and sleep disorders during the perioperative period. These issues seriously affect the quality of life and postoperative recovery of the patients. From the keywords in Figure 4, it is shown that the regulatory mechanism of TEAS is closely related to inflammatory response and stress reaction. Further exploration of the original literature revealed that TEAS has a modulating effect on a series of diseases and syndromes during the perioperative period, which is closely related to its effects on the neurologic, immune, and endocrine systems.

### 6.1. Postoperative pain

#### 6.1.1. Modulation of endogenous analgesic system-related factors

5-Hydroxytryptamine (5-HT), as an endogenous active substance, is involved in pain modulation both in the central and peripheral systems.^[21]^ Monoaminergic neurotransmitters, such as epinephrine and norepinephrine, mediate pain signaling in the dorsal horn. Additionally, inhibiting the 5-HTergic pathway affects nociceptive signaling through the activation of 5-HT receptors.^[21]^ The application of TEAS alone during ureteral lithotripsy reduces postoperative pain, decreases postoperative analgesic consumption, and decreases the production of the pain-causing substances 5-HT and substance P.^[22]^ β-Endorphin, an opioid receptor agonist, can reduce nociceptive sensitization, inhibit the release of substance P, reduce local vascular permeability, and increase the content of β-endorphin to promote the body’s analgesic effect.^[23]^ TEAS combined with general anesthesia for radical mastectomy increased the content of β-endorphin, thereby enhancing the analgesic effect.^[24]^ In addition, opiorphin has a significant analgesic effect, driving the body’s opioid system to combat pain and enhancing the regulation of the analgesic effect of enkephalin.^[25,26]^ In one study, the level of β-endorphin increased during general anesthesia for breast cancer surgery, and postoperative opiorphin levels were significantly elevated after the application of TEAS.^[27]^

#### 6.1.2. Regulation of stress-related substances

The release of pro-inflammatory factors can lead to tissue damage, including inflammatory factors and tumor necrosis factor-alpha (TNF-α). Enhanced peripheral injurious afferent activity can directly increase neural excitability and play a role in initiating and maintaining enhanced pain states.^[21]^ TEAS had an inhibitory effect on inflammation in the systemic inflammatory response after percutaneous nephrolithotomy, with TNF-α and interleukin-6 (IL-6) levels decreasing after TEAS treatment compared to the sham TEAS group, as well as a decrease in analgesic drug consumption.^[28]^

Secretory immunoglobulin A (SIgA) is an important immunoglobulin A antibody found in exocrine fluids, primarily acting on mucous membranes.^[29]^ It protects the body from infection and promotes the relief of infectious inflammatory reactions.^[30]^ SIgA, along with salivary amylase (sAA) and cortisol, are specific indicators of stress.^[31,32]^ It has been found that sAA levels are positively correlated with pain intensity and pain-induced unpleasantness.^[33]^ Therefore, sAA can also be a noninvasive, sensitive, and objective indicator for pain evaluation.^[34]^ In general anesthesia anorectal surgery, TEAS achieves analgesic anesthesia by effectively promoting the release of analgesic substances in the body, such as SIgA, sAA, and cortisol, reducing stress levels during the surgery.^[27]^

### 6.2. Postoperative gastrointestinal dysfunction

#### 6.2.1. Factors associated with the regulation of gastrointestinal tract functions

Gastrin and ghrelin are excitatory gastrointestinal hormones. Gastrin primarily regulates the cyclic activity of myoelectric complex waves during the inner digestive period and promotes gastric emptying.^[35]^ Gastrin normally promotes gastric acid secretion and gastrointestinal mucosal growth by binding to appropriate receptors.^[36]^ During and after laparoscopic intestinal surgery with TEAS, serum gastrin concentrations were increased 24 hours postoperatively, accelerating the recovery of gastrointestinal function.^[37]^ Gastrin in peripheral blood promotes gastric acid secretion through a mechanism related to the vagus nerve and histamine synthesis and release, and its effect is proportional to its concentration.^[38]^ TEAS can shorten the time to the first postoperative bowel sounds and the first bowel movement in patients undergoing gastrointestinal surgery, effectively promoting the recovery of gastrointestinal function. This therapy can increase the concentration of plasma ghrelin and motilin postoperatively, thereby promoting the recovery of gastrointestinal functions.^[16]^

5-HT is an important signaling factor in the intestine. 5-HT in the gastrointestinal tract is involved in gastrointestinal motility, epithelial secretion, and vasodilatation, as well as neuroprotective and pro-inflammatory effects. 5-HT also plays a role outside the gastrointestinal tract.^[39]^ A study using TEAS to treat women undergoing cesarean-sections found that intraoperative and postoperative nausea and vomiting symptoms were relieved after treatment, and plasma 5-HT concentration was significantly reduced, suggesting that the improvement of nausea and vomiting in cesarean-section women may be related to the reduction of plasma 5-HT concentration.^[40]^

#### 6.2.2. Regulation of stress-related substances

Norepinephrine, C-reactive protein, TNF-α, IL-6, and other stress-related substances play a pivotal role in gastrointestinal dysfunction. In thoracoscopic surgery under general anesthesia, TEAS of PC6 reduces cortisol and norepinephrine levels, mitigates the stress response, and decreases the incidence of nausea and vomiting in patients.^[41]^ During the postoperative period of lung cancer, TEAS reduces the inflammatory level in the body, decreasing C-reactive protein, TNF-α, IL-6, and other inflammatory factors, thus providing gastroprotection.^[42]^

#### 6.2.3. Regulation of the autonomic nervous system

In the central nervous system, ghrelin either enhances or inhibits gastric acid secretion in association with vagal activity.^[16]^ The sympathetic-to-vagal ratio is an independent risk factor for predicting the development of nausea and vomiting in patients after catheterized arterial chemoembolization.^[43]^ Sympathetic excitability can be reflected by standard diviation of NN intervals (SDNN) and low frequency (LF) indices in heart rate variability, while high frequency (HF) and root mean square of successive differences (rMSSD) can highlight vagal excitability. A study examined the impact of TEAS on postoperative gastrointestinal function, including gastric motility, gastrin levels, and heart rate variability in patients undergoing gastrointestinal surgery, the HF and rMSSD indices were significantly higher in the TEAS group at 4 days postoperatively compared with the sham-operated group, suggesting that transcutaneous acupoint electrostimulation significantly increased vagal excitability in these patients.^[16]^ Additionally, abnormalities in LF/HF, SDNN, and rMSSD indices were found in patients with gastrointestinal dysfunction after cholecystectomy. Transcutaneous electrical acupoint stimulation reduced LF/HF, SDNN, and rMSSD, suggesting that it can reduce sympathetic excitation in the early postoperative period, maintain parasympathetic tension, and promote the recovery of gastrointestinal function.^[44,45]^ Moreover, vagal excitability was negatively correlated with the time needed to return to a normal diet, time to defecation, and norepinephrine levels. In contrast, sympathetic excitability was positively correlated with the time needed to return to a normal diet and the time to onset of flatulence.^[46,47]^

### 6.3. Postoperative cognitive dysfunction

#### 6.3.1. Regulation of brain protective and brain damage factors

Serum neuron-specific enolase (NSE) and S100 calcium-binding protein β (S100-β) are components of neuroglia and neuronal cells, respectively. When these cells are structurally disrupted, NSE and S100-β are released into the blood and cerebrospinal fluid, and changes in their concentrations can be measured to reflect the severity of brain injury. Microtubule-associated protein Tau helps maintain microtubule stability in axons.^[48]^ Research has demonstrated that abnormal levels of hyperphosphorylated Tau are associated with the formation of neurogenic fiber tangles in the brain parenchyma.^[49]^ In the brains of Alzheimer patients, there is a decrease in normal Tau protein and a large increase in abnormally hyperphosphorylated Tau protein.^[50]^

TEAS was performed before, during, and after cholecystectomy in elderly patients. The results showed that TEAS had a significant effect on cognitive function and the incidence of postoperative cognitive dysfunction (POCD) after cholecystectomy. The incidence of POCD and the level of serum S100-β were significantly reduced, suggesting that the mechanism of TEAS may involve inhibiting the release of serum S100-β to reduce the incidence of POCD and thus protect brain function. Similarly, after single-port thoracoscopic lobectomy, S100-β, NSE, and hyperphosphorylated Tau levels were significantly lower in the TEAS group than in the control group, and cognitive levels were higher in the TEAS group.^[51]^

Calcitonin gene-related peptide (CGRP) is a prevalent neuropeptide in the central nervous system, known for its neuroprotective effects against ischemia/reperfusion injury and inflammatory responses.^[52,53]^ Studies have shown that CGRP can improve learning and memory by inhibiting TNF-α and inducing insulin-like growth factor 1.^[54]^ Additionally, CGRP plays an important role in gut-brain axis communication.^[55]^ Evidence suggests that TEAS reduces intraoperative serum IL-6 and hypersensitive C-reactive protein (hs-CRP) levels, inhibits inflammatory responses, increases CGRP, and reduces the duration of postoperative cognitive impairment in older adults.^[56]^

#### 6.3.2. Regulation of inflammatory factors

Cerebral ischemia releases a range of pro-inflammatory cytokines. In an ischemic, hypoxic environment, overactivation of microglia mediates an inflammatory response that can disrupt the blood–brain barrier and promote the development of vascular cognitive deficits.^[57,58]^ Inflammatory markers not only reflect peripheral diseases but also cerebrovascular disease mechanisms related to dementia. Therefore, markers of inflammatory response can be used as parameters for diagnosing vascular cognitive impairment, determining the severity of the disease, and assessing the prognosis. TEAS reduces the incidence of postoperative delirium, and the decrease of TNF-α and IL-1β concentrations suggests that TEAS for postoperative cognitive dysfunction may be associated with suppression of inflammation.^[59]^

#### 6.3.3. Regulation of other hormones

Melatonin is an amine neuroendocrine hormone secreted by the human pineal gland, which is secreted rhythmically and has a variety of physiological functions, including regulating oxidative stress, immune response, and neuroprotection. Studies have shown that general anesthesia for abdominal surgery in elderly patients can induce olfactory deficits, cause cognitive dysfunction, and reduce postoperative melatonin levels.^[60,61]^ Additionally, sevoflurane can impair short-term olfactory memory capacity, which is associated with humoral mechanisms that regulate changes in plasma melatonin levels.^[62,63]^ The preventive effect of TEAS on impaired postoperative olfactory memory capacity in patients under general anesthesia with sevoflurane may be related to the promotion of plasma melatonin secretion by TEAS.^[64]^

### 6.4. Postoperative blood pressure

#### 6.4.1. Regulating the stress response

Endothelin (ET) has strong vasoconstrictive properties, and cortisol is a commonly used indicator of the stress state of the organism. In a study of TEAS-assisted surgical anesthesia, the TEAS intervention promoted postoperative hemodynamic stabilization and stabilized ET and cortisol levels. It reduced the stress response more effectively than the general anesthesia group.^[65]^

#### 6.4.2. Regulation of the autonomic nervous system

In heart transplantation, TEAS application significantly improved heart rate variability indices and augmented sympathetic and vagal indices in transplant recipients. Significant increases in systolic and diastolic blood pressure and mean arterial pressure were observed during postoperative recovery. It suggests that the mechanism by which TEAS regulates postoperative blood pressure may be closely related to the autonomic nervous system.^[66]^

### 6.5. Postoperative ischemia–reperfusion injury

#### 6.5.1. Regulation of vascular endothelial function

ET, nitric oxide (NO), and von Willebrand factor (vWF) play important roles in the impairment of vascular endothelial function after percutaneous coronary intervention (PCI). ET-1 and NO are crucial vasoconstrictor and vasodilator factors in the body, both playing essential roles in maintaining normal vascular endothelial function.^[67]^ When coronary endothelial cells are damaged, serum NO secretion decreases, and ET-1 secretion increases, leading to an imbalance in vascular endothelial function and promoting cardiac injury.^[68,69]^ vWF, present in and secreted by Weibel-Palade bodies of endothelial cells, acts directly at the site of vascular injury and is considered a biomarker of vascular endothelial dysfunction.^[70]^ The levels of ET-1 and vWF in the TEAS group were significantly lower than those in the sham TEAS group at 8 and 24 hours after the procedure. The serum NO level and endothelium-dependent vasodilatory function were significantly higher in the TEAS group, suggesting that TEAS during the perioperative period of PCI effectively improves vascular endothelial function, reduces post-interventional vascular endothelial injury, and protects the heart.^[71]^

#### 6.5.2. Modulation of the inflammatory response

The interaction of inflammatory response and endothelial dysfunction promotes cardiovascular issues. hs-CRP, a specific indicator of inflammation, interacts with vascular endothelial and other cells to accelerate vascular inflammatory response, leading to plaque rupture within the coronary arteries.^[72,73]^ Clinical studies have confirmed that IL-10 is an anti-inflammatory cytokine that protects against the body’s inflammatory response, reduces intimal hyperplasia following vascular injury, and inhibits endovascular inflammation. In contrast, IL-6 is a pro-inflammatory cytokine that induces the liver to produce acute-phase proteins and fibrinogen, exacerbating coronary artery inflammation and accelerating thrombosis.^[74,75]^ Maintaining a balance between pro-inflammatory and anti-inflammatory cytokines during PCI surgery is crucial for attenuating the inflammatory response and protecting the myocardium. In a study of PCI surgery, the levels of hs-CRP, MMP-9, and IL-6 were significantly lower in the TEAS group than in the sham TEAS group at 8 and 24 hours postoperatively, while IL-10 levels were significantly higher, suggesting that TEAS reduces serum inflammatory factor levels after PCI.^[71]^

### 6.6. Postoperative immunosuppression

#### 6.6.1. Regulation of T lymphocyte subsets

Immune function is essential for postoperative recovery. Both intraoperative anesthetics and postoperative pain can contribute to immunosuppression.^[76]^ The recovery of immune function significantly impacts the prognosis and survival of surgical patients.^[77]^ T-lymphocyte subsets (CD3^+^, CD4^+^, and CD8^+^) are key indicators of cellular immunity.^[78]^ Elevated levels of these subsets suggest immunocompromise.^[79]^ The CD4^+^/CD8^+^ ratio provides insight into the body’s overall immune status. TEAS can help maintain the balance of T-lymphocytes and regulate cytokine expression, thereby reducing postoperative immune injury in lung cancer patients. In laparoscopic gastric cancer surgery, TEAS was found to increase levels of CD3^+^ and CD4^+^ cells and improve the CD4^+^/CD8^+^ ratio, while decreasing CD8^+^ cell levels.^[80]^

Activation and differentiation of T lymphocytes are crucial for effective anti-infective and antitumor immune responses.^[81]^ Imbalances among T helper cell subsets—such as Th1, Th2, Th17, and regulatory T cells—can lead to immune disorders.^[82]^ Th1 cells produce IFN-γ and IL-2, which are vital for anti-inflammatory and antitumor activities,^[83,84]^ while Th2 cells release pro-inflammatory cytokines, primarily IL-10.^[85,86]^ Th17 cells exhibit potent pro-inflammatory properties by secreting IL-17, IL-22, and TNF-α. Additionally, regulatory T cell deficiency or abnormal function can lead to autoimmune diseases.^[87]^ TEAS administration raised the percentage of Th1 and Th17 cells and boosted protein levels of IL-2 and IFN-γ, while lowering the percentage of Th2 cells and IL-10 expression. Additionally, it increased the expression of transcription factors in T helper cells, including T-bet, RORγT mRNA, and GATA-3 mRNA, thereby partially mitigating postoperative immune suppression in cancer patients.^[12,88]^

### 6.7. Postoperative sleep disorders

#### 6.7.1. Regulation of oxidative stress

Stress is linked to poor sleep quality.^[89]^ Traumatic stress triggers excessive production of reactive oxygen species (ROS), leading to oxidative stress, which can contribute to sleep disorders.^[90]^ TEAS can elevate the levels of Nrf2, GPX3, and SOD on the 1st and 3rd days after surgery, lower cortisol levels, and enhance sleep quality. This indicates that TEAS may improve sleep through the activation of the Nrf2 signaling pathway and a reduction in stress response.^[91]^

#### 6.7.2. Regulation of inflammatory cytokines

Inflammatory cytokine activation is an important change caused by sleep disorders. In turn, inflammatory cytokines can lead to altered central nervous system activity and disruption of sleep regulation through neural and cellular mechanisms.^[92,93]^ The accumulation of ROS induced by oxidative stress plays a crucial role in inflammatory responses.^[94]^ Nrf2 activation can indirectly inhibit the transcription of pro-inflammatory cytokines.^[95]^ Additionally, eliminating ROS has been shown to reduce inflammation in Nrf2-deficient mice.^[96]^ As mentioned earlier, TEAS has a regulatory effect on oxidative stress and Nrf2 activation. Therefore, TEAS downregulates IL-6 expression in the early postoperative period, which may also be related to the activation of the Nrf2/ARE pathway.^[91]^

## 7. Hot spots for transcutaneous acupoint electrical stimulation applications

From the #5 acupoint clustering and the frequency of keyword occurrence, PC6, ST36, and LI4 are common acupoints used for treatment. In the #11 LI4 clustering, ST36 and LI4 are closely related to the improvement of quality of life and reduction of adverse reactions. Previous studies have shown that stimulation of these 3 acupoints induces the midbrain, hypothalamus, and spinal cord to produce and release endorphins, dynorphins, and enkephalins, which exert analgesic effects.^[20]^ Acupoint stimulation of LI4, PC6, and ST36 has been found to improve analgesia and reduce perioperative suppression of immune function in patients undergoing radical mastectomy for breast cancer.^[12]^ The same type of nerves may innervate the LI4 and PC6 points in a certain region.^[97]^ ST36 slows morphine analgesic tolerance and reverses morphine motor sensitization.^[98]^ ST36 is also involved in the expression of substance P to exert analgesic effects.^[99]^

## 8. Shortcomings and prospects

Currently, TEAS clinical studies still need to improve: (1) among the 1296 Chinese and English articles included from 1988 to 2024, large-scale, multicenter, double-masked, RCTs are still rare. About two-thirds of the literature is published in noncore journals in China, indicating that the quality of the clinical evidence needs to be strengthened. (2) In terms of core authors, cooperation modes, and research institutions, although a network of core authors has been formed and has some influence in China, the cooperation modes are relatively homogeneous, and the number of different institutions involved is small. (3) Research on clinical mechanisms should be further strengthened. From the keyword co-occurrence analysis and keyword clustering mapping, there are scattered keywords such as *inflammatory factors*, *interleukins*, and *heart rate variability*. However, the nodes are so small that they cannot be displayed. It indicates that research on the clinical mechanisms of TEAS, especially the inflammation-related mechanisms, is still in the preliminary stages and lacks depth. (4) From the perspective of hot spots and trends in clinical research on TEAS, although transcutaneous acupoint stimulation can promote perioperative recovery to a certain extent, there is no uniform international standard for the intensity and duration of acupoint stimulation. Moreover, there needs to be more scientific standardization for the selection criteria of acupoint preparation, especially regarding appropriate stimulation points and parameters for different surgeries. These issues limit the application and popularization of acupoint stimulation in the perioperative period.

Future TEAS research can focus on the following aspects: (1) strengthen inter-institutional cooperation and actively carry out large-scale, multicenter, double-masked clinical RCTs, prioritize research institutions in cities such as Beijing, Shanghai, and Zhejiang. (2) Research related to TEAS in advantageous diseases, suitable stimulation points, and stimulation technology parameters should be actively conducted to promote the process of TEAS clinical standardization. (3) Actively pursue research on the clinical mechanisms of TEAS. Some clinical trials on TEAS have begun to study its regulatory mechanism on heart rate variability and inflammation, but the research depth still needs to be deeper. Heart rate variability is the most valuable noninvasive test to reflect the function of the autonomic nervous system.^[100]^ Meta-analyses have demonstrated that TEAS regulates autonomic nervous system activity by activating the vagus nerve, proving that its efficacy is comparable to traditional acupuncture.^[100]^ Increasing evidence suggests that the autonomic nervous system can modulate inflammatory reflexes, with a positive correlation between some heart rate variability indices and inflammatory markers.^[101]^ There is a close physiological relationship between the vagus nerve and the inflammatory process, with vagus nerve-mediated heart rate variability indices possibly related to the reduction of inflammation through the cholinergic anti-inflammatory pathway.^[102]^ Therefore, in addition to investigating the effects of TEAS on classical inflammatory pathways, exploring the therapeutic mechanisms of TEAS from perspectives such as heart rate variability is a worthwhile direction for current research.

## Author contributions

**Conceptualization:** Mengqi Li.

**Data curation:** Xiaobo Jiang, Xiangmu Gai.

**Funding acquisition:** Hongfeng Wang.

**Investigation:** Xiaobo Jiang, Xiangmu Gai.

**Methodology:** Mengqi Li.

**Project administration:** Mengyao Dai.

**Resources:** Mengyuan Li.

**Supervision:** Yanxin Wang.

**Visualization:** Mengyuan Li.

**Writing – original draft:** Mengqi Li.

**Writing – review & editing:** Mengqi Li, Xiaobo Jiang, Xiangmu Gai, Mengyao Dai, Mengyuan Li, Yanxin Wang, Hongfeng Wang.
